# The Complex Intracellular Lifecycle of *Staphylococcus aureus* Contributes to Reduced Antibiotic Efficacy and Persistent Bacteremia

**DOI:** 10.3390/ijms25126486

**Published:** 2024-06-12

**Authors:** Cecilia F. Volk, Richard A. Proctor, Warren E. Rose

**Affiliations:** 1 Pharmacy Practice and Translational Research Division, School of Pharmacy, Pharmacy University of Wisconsin-Madison, Madison, WI 53705, USA; cvolk2@wisc.edu; 2 Department of Medicine, School of Medicine and Public Health, University of Wisconsin-Madison, Madison, WI 53705, USA; 3 Department of Medical Microbiology and Immunology, School of Medicine and Public Health, University of Wisconsin-Madison, Madison, WI 53705, USA

**Keywords:** resistance, small colony variant, relapse, recurrence, bloodstream

## Abstract

*Staphylococcus aureus* bacteremia continues to be associated with significant morbidity and mortality, despite improvements in diagnostics and management. Persistent infections pose a major challenge to clinicians and have been consistently shown to increase the risk of mortality and other infectious complications. *S. aureus*, while typically not considered an intracellular pathogen, has been proven to utilize an intracellular niche, through several phenotypes including small colony variants, as a means for survival that has been linked to chronic, persistent, and recurrent infections. This intracellular persistence allows for protection from the host immune system and leads to reduced antibiotic efficacy through a variety of mechanisms. These include antimicrobial resistance, tolerance, and/or persistence in *S. aureus* that contribute to persistent bacteremia. This review will discuss the challenges associated with treating these complicated infections and the various methods that *S. aureus* uses to persist within the intracellular space.

## 1. Introduction

Antibiotic resistance in *Staphylococcus aureus* continues to pose significant risk to the healthcare landscape due to limited antimicrobial options [1]. However, persistent *S. aureus* infections present a major challenge to clinicians during the treatment of “susceptible infections” in patients. This is particularly true for persistent *S. aureus* bacteremia, characterized by repeatedly positive *Staphylococcus aureus* cultures in the bloodstream despite apparently appropriate antibiotic therapy [2]. This condition is associated with increased morbidity, mortality, and healthcare costs, making its management a priority in clinical practice [2,3]. From this perspective, we overview the problem of persistent bacteremia caused by *S. aureus* and highlight the intracellular proclivity of the organism as a mechanism for survival in the host and defense against antibiotic treatments. 

## 2. The Clinical Challenges of Persistent *Staphylococcus aureus* Bacteremia (SAB)

Persistent SAB is defined by repeat positive blood cultures over multiple days; however, the number of days required for an infection to be considered persistent is widely debated, with more contemporary definitions shifting towards earlier cutoffs that support early switching to alternative regimens [2]. This has aligned with rapid advances in molecular organism identification (i.e., MALDI-TOF and others) and automated antimicrobial susceptibility testing [4,5]. However, because this definition is not standardized, it therefore allows for a range of in-practice interpretations of when to consider changes in antibiotic therapy [2]. Traditionally, and informed from historical clinical trials, the definition of persistent bacteremia derived from the average duration of bacteremia among clinical trials of antibiotic therapy—around 7 days [6,7]. Since then, several studies have suggested earlier definitions of persistent SAB (the 3-5-day duration of positive cultures) due to improved outcomes noted with shorter durations of bacteremia [3,8,9]. For example, we argued persistent or prolonged bacteremia be considered as the >4-day duration of positive cultures based upon the observed increase in mortality and cytokine markers associated with this threshold from regression analysis [8,10]. This time frame corresponds with the final identification and susceptibility profile of *S. aureus* obtained from blood cultures accounting for time to growth in blood culture vials, identification by molecular methods, and phenotypic susceptibility. However, recently, Holland et al. proposed considering persistent bacteremia earlier, after one calendar day, for the further diagnostic evaluation and consideration of antibiotic failure for implementing alternative treatment regimens [2]. This suggestion is supported by a study by Kuehl et al., which found that patients who had positive blood cultures beyond 24 h of antibiotic therapy were at a higher risk of mortality and metastatic infections [11]. The overwhelming clinical evidence implies an excessive morbidity and mortality risk of persistent bacteremia for patients and recommends a low threshold for early antibiotic changes for rapid bacteremia clearance. 

## 3. Treatment Approaches for Persistent SAB 

Several treatment options still exist for *S. aureus*; however, the treatment of complicated bacteremia is limited to only a handful of approved agents. This includes anti-staphylococcal β-lactams (nafcillin or oxacillin) and targeted cephalosporins (i.e., cefazolin) for MSSA, while vancomycin or daptomycin are typically reserved for MRSA. Recently, ceftobiprole received FDA approval for MSSA and MRSA bacteremia and endocarditis based on a randomized controlled trial demonstrating its non-inferiority to daptomycin [12], and ceftaroline has been used similarly without indication [13,14,15]. Following induction with these antibiotics, other agents are commonly used as step-down therapy, including linezolid (IV/PO), the lipoglycopeptides dalbavancin and oritavancin, and many oral options including β-lactams, trimethoprim–sulfamethoxazole, and clindamycin [16]. Clearly, there is a diverse range of options for the treatment of SAB. However, these options considerably narrow when confronted with MRSA or in patients with persistent bacteremia [3,16]. 

The successful management of persistent SAB requires a comprehensive approach that addresses both host and pathogen factors. Initial management involves timely and appropriate antibiotic therapy guided by antimicrobial susceptibility testing [17]. However, given the potential for antibiotic resistance and/or tolerance, combination therapy with agents targeting different bacterial pathways has been used successfully to improve bacterial clearance [3,16]. This is of highest concern in persistent MRSA bacteremia, where treatment options are extremely limited once the standard-of-care agents are deemed unsuccessful. Any randomized controlled trial of SAB implements a non-inferiority design [12,18,19,20,21]. No randomized controlled trial exists to understand the optimal treatment of persistent SAB; therefore, treatment approaches are guided by observational and retrospective case–control studies. From this evidence, treatment paradigms have been proposed to consider how to address persistent MRSA bacteremia and include combination therapy with priority towards daptomycin plus ceftaroline followed by daptomycin plus an antistaphylococcal beta-lactam, and then, finally, vancomycin plus a hydrophilic beta-lactam to reduce the risk of nephrotoxicity (e.g., cefazolin, ceftaroline) [16]. In addition to antibiotic therapy, the removal of potential sources of infection, such as infected intravascular devices or sites of metastatic infection, is essential. This may involve the removal or exchange of indwelling catheters, the debridement of infected tissues, or the surgical drainage of abscesses. Close monitoring for complications, such as infective endocarditis or metastatic infections, is also imperative during persistent bacteremia [16]. 

While antibiotic therapy is crucial for a successful outcome in SAB, host factors also play a significant role in the persistence of *S. aureus* and must be carefully evaluated and managed [22]. Underlying conditions that compromise host immune function, such as diabetes mellitus or immunosuppressive therapy, must be evaluated and managed when possible. Some host characteristics can be either modifiable or non-modifiable. For example, patients with diabetes can have blood glucose managed within the normal range; however, the underlying immunologic suppression and ischemia from this conditional is unchanged, which prevents the complete eradication of complicating and contributing factors in these patients. Optimal supportive care, including nutritional support and management of comorbidities, is also essential to enhance host defenses and improve outcomes. However, the host–pathogen dynamic in SAB remains poorly understood [23,24,25]. This is evident from the failed vaccination attempts in human trials aimed at preventing *S. aureus* invasive infections despite promising animal model data [23]. Only recently has the underlying host immunologic pathology occurring at the onset and during SAB begun to be understood. From these studies, it is noted that profound cytokine imbalances exist in patients with SAB [8,23,24,26,27]. The pro-inflammatory cytokine response appears to be important during early initial response and clearance bacteremia (e.g., IL-1β, IL-2, IL-6, TNF-α, and glutamine) [8,27]. In terms of survival outcome, high levels initially and throughout the duration of bacteremia appear to be detrimental either to excessive pro-inflammatory response (e.g., IL-6 and TNF) or host-immune paralysis (e.g., IL-10, IL-6, IL-17, CCR2, T4, adiponectin) [10,23,28,29]. Related to persistent bacteremia, a dampening of the pro-inflammatory cytokines IL-1β and TNF-α has been linked to the duration of bacteremia as a result of the pathogens’ response to antibiotic exposure and ability to evade host defenses through multiple mechanisms [8,10,27,28]. 

An additional factor that often complicates treatment is the diversity of toxins and virulence factors that *S. aureus* can produce. Multiple studies have linked differential toxin expression to patient outcomes in a variety of *S. aureus* infection types, including bacteremia [23,30,31,32]. Importantly, elevated innate levels of certain anti-toxin antibodies can be protective against severe disease [33,34,35]. These important observations have led to the evaluation of anti-toxin antibodies in early-stage clinical trials, including AR-301, an anti-α-toxin monoclonal antibody tested as an adjunctive therapy in patients with hospital-acquired bacterial pneumonia caused by *S. aureus* [36]. This trial only included 48 patients but found shorter durations of ventilation and faster microbiologic eradication in the patients who received the antibody. Antibiotics with anti-toxin effects are also being evaluated as adjunctive therapies for SAB. Specifically, clindamycin, a protein synthesis inhibitor that inhibits toxin production, was evaluated via an open-label randomized controlled trial [37]. This trial included 34 patients with severe *S. aureus* infections and found a lower mortality rate in patients receiving adjunctive clindamycin (0% vs. 24%). This treatment is currently being evaluated further in the *S. aureus* Network Adaptive Platform (SNAP) randomized controlled trial [38]. These preliminary studies highlight the likely importance of toxin inhibition in managing SAB.

In summary of its clinical importance and treatment challenges, persistent SAB poses a significant clinical problem, characterized by ongoing bloodstream infection despite appropriate therapy. Successful management requires a multifaceted approach that addresses both pathogen and host factors. Timely and appropriate antibiotic therapy, the removal of potential sources of infection, the optimization of host defenses, and efforts to prevent recurrent infections are key components of management. Ultimately, persistent SAB represents a complex clinical syndrome that requires a multidisciplinary approach involving infectious disease specialists, clinical microbiologists, pharmacists, nurses, and other healthcare professionals. Collaboration between specialties is essential to ensure timely diagnosis, appropriate treatment, and ongoing management of this challenging condition. However, the identification of why *S. aureus* persists from an organism, host, and mechanistic level remains key to solving this issue. 

## 4. Small-Colony-Variant *S. aureus* Results in Reduced Antibiotic Efficacy and Infection Persistence

Although the reduction in the duration of SAB has been a noble goal over the last three decades, the mechanistic understanding of how and why *S. aureus* persists when susceptible to antibiotic therapy remains elusive. There are several proposed mechanisms contributing to this phenomenon. *S. aureus* has metabolic adaptations to survive and hide from the immune systems of humans. One of the most studied phenotypes contributing to persistence is the small colony variant (SCV) of *S. aureus*. This is a unique phenotype characterized by distinct morphological and growth characteristics [39]. SCVs contribute to the persistence of many invasive infections including bacteremia, pneumonia, and osteomyelitis, among others, and SCVs typically exhibit slow growth rates and form small, non-pigmented colonies on blood agar [39,40,41]. SCVs remain difficult to detect using standard laboratory methods for surveillance studies due to the lack of dedicated screening methods and noted instability of the phenotype, but they may contribute to up to 30% of invasive *S. aureus* infections [40].

Small colony variants were first described in 1911 as a slow-growing subpopulation [42] and later noted for their difference in “color, texture, and viscidity” [43]. Early research identified SCV occurrence correlated to harsh environmental conditions such as high salt content [44]. The clinical impact of SCVs arose in the early 1990s by identifying this phenotype as a cause of persistent and relapsing *S. aureus* infections [39]. Since this time, the understanding of the clinical relevance and emergence of SCVs has appeared through genetic mutations or adaptive responses to various environmental stresses during infection in patients, such as exposure to antibiotics or host immune defenses. These mutations often result in alterations in metabolic pathways, leading to changes in bacterial physiology and virulence [45]. Clinical manifestations of SCV infections vary depending on the site of infection (e.g., endocarditis vs. osteomyelitis) and underlying host factors [45,46]. 

A hallmark feature of SCVs is their reduced susceptibility to antibiotics, particularly aminoglycosides and cell wall-active agents like β-lactams [40,47]. This reduced susceptibility is attributed to decreased metabolic activity and alterations in membrane potential, which impair antibiotic uptake and efficacy through reductions in the production of active targets such as peptidoglycan and penicillin-binding proteins [45,48,49,50]. In addition to resistance to these key antibiotics, particularly β -lactams for MSSA, SCVs have reduced susceptibility to standard-of-care agents for MRSA—vancomycin—through reduced peptidoglycan formation and daptomycin since active membrane potential is required for activity [40,48,51]. The management of SCV infections is complex and often involves prolonged courses of antibiotic therapy tailored to the specific susceptibility profile of the isolate. Antimicrobials with high intracellular uptake, such as fluoroquinolones, may be preferred, when susceptible, to treat the intracellular reservoir [48]. Combination therapy may be necessary to enhance bacterial clearance and prevent the emergence of resistance, but the optimal combination for SCV treatment is not established [47,48]. The lipoglycopeptide oritavancin, which has the dual inhibition of peptidoglycan and cell membrane synthesis, may have specific activity against *S. aureus* SCVs [52,53]. Natural products also are being explored against SCVs, including tomatidine and its derivatives, which have been noted to block F_0_F_1_ATPase [54]. These molecules also synergize with aminoglycosides and prevent SCV formation [55]. 

## 5. Antimicrobial Resistance, Tolerance, and Persistence in *S. aureus* Contribute to Persistent Bacteremia

**Figure 1:**
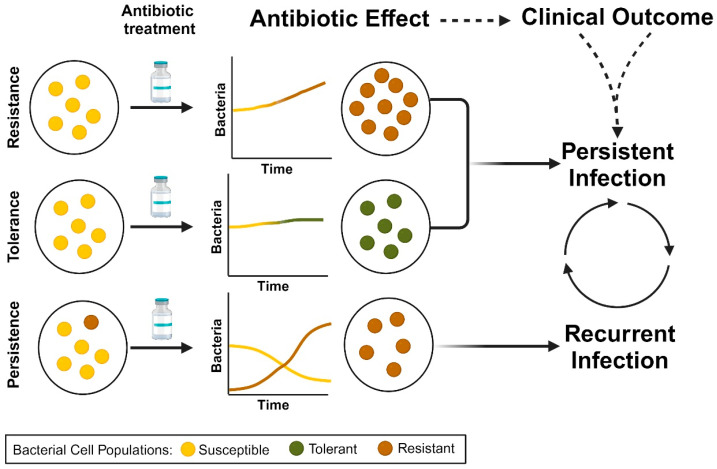
Concept of antimicrobial resistance, tolerance, and persistence in *S. aureus* and potential impact on treatment outcomes. Different bacterial populations are represented by the color scheme (gold, susceptible; green, tolerant; reddish-brown, resistant).

While major attention is rightfully paid toward antimicrobial resistance in *S. aureus*, the occurrence of antimicrobial tolerance and persistence also represents significant issues for antimicrobial therapy [56,57,58,59]. Observations of antimicrobial tolerance and persistence in *S. aureus* represent a growing area of interest and investigation [60,61]. Antimicrobial tolerance is distinct from antimicrobial resistance in that tolerant strains appear to be susceptible upon MIC testing in liquid culture. The nongrowing or slowly growing bacterial population remains viable and can “tolerate” or survive temporary antimicrobial exposure [62] (Figure 1). These tolerant populations may enter a dormancy state or reduce cellular processes to survive antimicrobial pressure through reductions in active targets (e.g., reduced cell-wall replication) [60]. Antimicrobial tolerance may develop through either a genetic mutation triggered by environmental conditions (e.g., reduced oxygen or nutrients leading to mutation(s) in TCA cycle genes) or antibiotics (single-point mutations) [58,60,63]. In contrast to tolerant cells, persisters are a subset of the bacterial population that can survive high concentrations of antimicrobial exposure. During treatment, the majority of the population may be killed by therapeutic concentrations, leaving the “persister cells” remaining to subvert antimicrobial treatment (Figure 1) [60]. Similar to tolerant cells, persisters may enter a state of dormancy and altered metabolism during this phase [64,65,66]. Once the antimicrobial exposure is removed, these cells may begin to replicate, leading to infection recurrence [64]. While SCVs described previously in detail can also be tolerant and persisters to antibiotics, these phenomena are not restricted to SCVs. Different mechanisms for SCVs and persisters exist, but a commonality between these two phenotypes is the decreased ability to make ATP [67].

An added layer to antimicrobial tolerance and persistence is the ability of *S. aureus* to produce robust biofilms [68,69]. These are complex communities of bacteria encased within a self-produced extracellular matrix consisting of polysaccharides, proteins, and extracellular DNA [70]. Biofilms provide protection against antibiotics and host immune responses, allowing bacteria within the biofilm to survive and persist despite antibiotic exposure [71,72]. Both tolerant bacterial populations and persister cells occur deep within the biofilm, and treatment is further compromised by a lack of antibiotic penetration through the extracellular matrix [73,74]. The low oxygen environment of biofilms combined with high bacterial burden may also reduce ATP and membrane potential [75]. In bacteremia, biofilms are known to play an important role in the pathology of the infection sources including endocarditis, prosthetic devices, and osteomyelitis [69].

Antimicrobial tolerant and persistent *S. aureus* pose a significant clinical challenge as they can lead to treatment failure, recurrent infections, and the spread of antibiotic resistance [60,76]. Arguably, antimicrobial tolerance is responsible for persistently positive SAB (consecutive days of bacteremia), while persister cells are responsible for bacteremia recurrence (positive cultures after day(s) of negative cultures) (Figure 1). The effective management of antimicrobial tolerant/persistent *S. aureus* infections often requires a multifaceted approach, including the use of combination antibiotic therapy, the removal of biofilm-associated infections, and the development of novel antimicrobial strategies to target tolerant/persistent bacterial populations [60]. However, the challenge of identifying these strains is a significant barrier for the early recognition and initiation of targeted therapy. Currently, the assays to test for tolerant/persistent *S. aureus* are experimental, and identifying these strains only occurs through the examination of an altered colony phenotype (e.g., SCV) or infection progression despite antibiotic therapy [56]. Understanding potential reservoirs for *S. aureus* tolerant populations and persistent cells is important for improving the response to antimicrobial therapy. 

## 6. Proclivity of *Staphylococcus aureus* for Intracellular Growth and Persistence

Although *S. aureus* is traditionally considered an extracellular pathogen, there is now substantial evidence supporting the presence of an intracellular niche for this organism [77]. Using in vitro assays, *S. aureus* has been shown to survive within a wide variety of cell types, including epithelial cells, endothelial cells, fibroblasts, osteoblasts, osteocytes, keratinocytes, macrophages, and neutrophils [64,78,79,80,81,82,83]. These bacteria have often been shown to be in a semi-dormant, non-culturable, small-colony-variant state [83,84]. Evidence of intracellular *S. aureus* persistence as a cause of human disease has been harder to prove, although a few studies have successfully done so. The presence of intracellular *S. aureus* during human infections has now been demonstrated in patients with recurrent rhinosinusitis, tonsillitis, osteomyelitis, and bacteremia [83,85,86,87,88,89]. Importantly, these studies all examined patients with recurrent or chronic infections. This evidence highlights intracellular survival as an important part of the lifecycle of persistent *S. aureus* infections.

The evidence for intracellular *S. aureus* as a contributing mechanism of persistent *S. aureus* infections is evident by the research and understanding of SCVs in this environment. *S. aureus* SCVs have a noted preference for increased intracellular uptake because of bacterial surface proteins that bind host receptors, such as epithelial cells and macrophages [67]. This intracellular lifestyle enables SCVs to evade innate host defenses and antibiotics, leading to chronic or recurrent infections that are challenging to eradicate, contributing to persistent or relapsing infections. However, this proclivity for intracellular invasion is not restricted to SCVs, and *S. aureus,* regardless of colony phenotypes, becomes an intracellular pathogen. 

## 7. Intracellular *S. aureus* Reduces Immune Recognition and Activation

*S. aureus* that persists intracellularly does so by avoiding the normal immune system activation during acute infection. Much of the typical immune activation is regulated by the accessory gene regulator (*agr*) system. *Agr* is a quorum-sensing system that regulates the expression of many toxins and other inflammatory factors [90] including α-toxin, which is known to induce inflammation and cell death when produced in an intracellular environment [91,92,93]. Several studies have tested clinical isolates from persistent *S. aureus* infections and found that these strains tend to have an *agr* deficiency [67,94,95,96]. Similarly, SCVs have been shown to have decreased *agr* expression [67]. In vitro studies have demonstrated that these *agr*-deficient strains have a higher rate of cellular uptake but lower induction of inflammatory responses [90,97]. SCVs have demonstrated lower production levels of IL-1β, IL-6, and IL-12 than wild-type *S. aureus* after the infection of epithelial cells [98]. In fact, a mouse model of chronic osteomyelitis infection has demonstrated that inflammatory markers return to normal levels during the infection, despite the persistent bacteria remaining intracellularly [99]. In addition, a study of patients with chronic rhinosinusitis with nasal polyps found no increase in the number of eosinophils, lymphocytes, and neutrophils in tissue samples, despite the presence of intracellular *S. aureus* [86]. Overall, this decreased immune activation allows for *S. aureus* to hide in the intracellular environment and largely avoid the host immune system. 

There are multiple methods that *S. aureus* can utilize to survive in an intracellular environment. For *S. aureus* to enter host cells, the upregulation of alternative sigma factor B (SigB) is required. This regulatory system modulates the *S. aureus* stress response through the transcription of genes that control resistance to heat and oxidative and antibiotic stresses and contributes to the SCV phenotype [67]. After uptake into host cells, *S. aureus* is typically taken up into phagosomes. This is consistent for both phagocytic cells and non-professional phagocytes such as epithelial cells, osteoblasts, and fibroblasts [92,100,101,102]. The phagolysosome environment has many antibacterial properties, including an acidic pH of 4.5 [25]. *S. aureus* is capable of not only tolerating an acidic environment, but also deacidifying the environment through the production of ammonia [103]. Although the downregulation of *agr* is important for initial cell invasion, as noted above, the intracellular acidic environment then enhances the expression of the *agr* system, increasing the survival of *S. aureus* intracellularly [104,105,106]. This effect is likely strain specific [107,108]. *S. aureus* is also capable of protecting itself from defensins and other antimicrobial peptides within the phagosome environment. The GraRS system leads to the upregulation of multiple peptide resistance factor (MprF) which confers protection from host defense peptides by enhancing the lysinylation of phosphatidylglycerol to the outer portion of the cellular membrane [109]. Similarly, the *dlt* operon (*dltABCD*) and *oatA* lead to alterations in membrane teichoic acids and peptidoglycan, respectively, which lead to protection from the phagosome environment [109,110]. Finally, *S. aureus* may produce protective enzymes (catalase, superoxide dismutase, etc.) to protect themselves from reactive oxygen species produced within the phagosome [111,112]. An increase in SodM, an enzyme responsible for detoxifying reactive oxygen species, was discovered in *S. aureus* isolates from patients with persistent cystic fibrosis infections [113]. In vitro assays have determined that this increase is noted specifically after the internalization of *S. aureus* into airway epithelial cells [114].

Despite these adaptations to the harsh phagosome environment, many types of *S. aureus* will exit the phagosome as a method to increase survival. This phagosomal escape is an *agr*-mediated process, as *agr* mutant strains are not capable of escaping to the cytosol. An increase in *agr* expression has been measured just prior to phagosomal escape [115,116]. Phenol-soluble modulins (PSMs), *agr*-dependent cytotoxic peptides that are activated during the stringent response, have been proven to play an important role in escape into the cytosol [115,117,118,119,120]. The concentration of PSMs has been similarly shown to increase just prior to *S. aureus* phagosomal escape, which has proven to be a required component in this process [117,121]. Other *S. aureus* escape factors, including a non-ribosomal peptide synthetase and a Tet38 efflux pump, have been identified, but their mechanisms are less clear [122,123]. In addition to these mechanisms, *S. aureus* may escape phagosomal destruction during overwhelming infection. In these situations, host cells are not able to tackle the high bacterial burden and become exhausted, creating an intracellular niche for *S. aureus* replication [124,125,126]. After phagosomal escape, replication within the cytosol leads to host cell lysis and the release of *S. aureus* [108]. These released cells may be re-phagocytosed, leading to a cycle that maintains a portion of intracellular *S. aureus* [126,127]. This maintenance of intracellular *S. aureus* can also contribute to the spreading of infection to other host sites, when mobile phagocytes are infected [126,128]. 

The above survival mechanisms appear to contradict the fact that persistent infections are often caused by *agr*-deficient *S. aureus* strains, as these strains would be unable to escape the phagosome due to lack of PSMs [67,94,95,96]. Indeed, these *agr*-deficient strains employ different survival methods that do not require PSM-mediated phagosomal escape. Siegmund et al. determined that PSM-deficient *S. aureus* has a higher overall survival rate within endothelial cells [129]. The surviving bacteria were co-localized with LC3, a marker of autophagy, indicating that these bacteria can survive within vesicles, likely by interfering with lysosomal recruitment and the autophagy process. Unlike the process of phagosome escape and host cell lysis presented above, this PSM-independent process allows for a consistent niche of intracellular growth. 

## 8. Anaerobic Metabolism of *S. aureus* Correlates to Intracellular Growth

**Figure 2:**
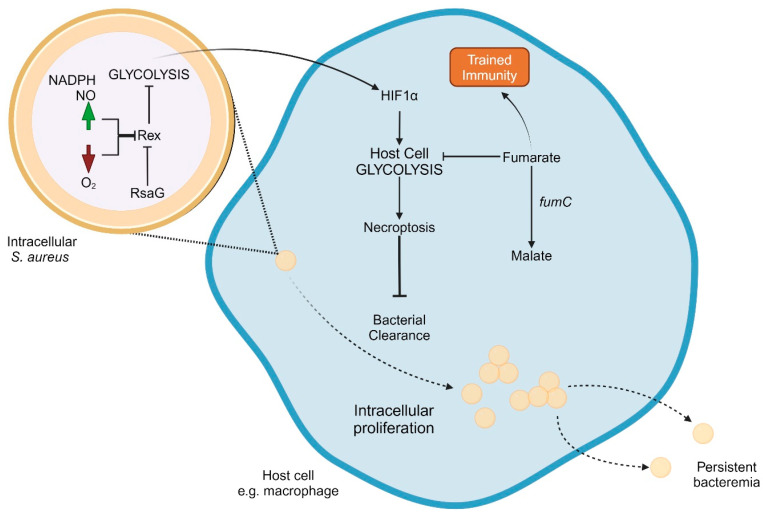
Model of intracellular lifecycle and anaerobic metabolism of *S. aureus* contributing to persistent infection.

There is a growing body of evidence to suggest that *S. aureus* utilizes anaerobic metabolism during persistent intracellular infections. Figure 2 presents a working model of key interactions of intracellular *S. aureus* metabolism and host factors leading to persistence. *S. aureus* SCVs have demonstrated a decrease in TCA cycle activity and a corresponding increase in glycolytic activity compared to wild-type strains, an effect that is consistent across all auxotrophic types of SCVs [50]. This effect, however, does not seem to be restricted to *S. aureus* with the SCV phenotype. One study conducted whole-genome sequencing of 206 MRSA isolates from patients with persistent bacteremia [130]. The authors found frequent mutations in genes involved in the TCA cycle (*citZ* and *odhA*). Mutations in these genes have been linked to antibiotic tolerance [65,66], which may help explain persistence despite adequate antimicrobial treatment. Similarly, *S. aureus* isolates from persistent cystic fibrosis infections have the downregulation of many proteins involved in amino acid metabolism, fatty acid metabolism, and energy metabolism [113,131]. These studies also found a reduction in the expression of proteins in the phosphoenolpyruvate carbohydrate phosphotransferase systems, which are responsible for the transport of glucose into the bacterial cell, a necessary precursor to glycolysis.

The redox regulator Rex inhibits important genes involved in anaerobic metabolism, including lactate dehydrogenase and alanine dehydrogenase. Rex plays an important role in activating anaerobic metabolism in response to intracellular uptake or in response to high concentrations of NADH or NO. RsaG is an sRNA that inhibits the expression of Rex [132,133]. RsaG is expressed in response to high levels of glucose-6-phosphate, as frequently found in the host cell cytosol or mucoid secretions. An increase in RsaG has been noted during in vitro internalization assays using both myoblasts and macrophages, as well as after exposure to mucus-secreting epithelial cells. This increase in RsaG therefore triggers a switch to anaerobic metabolism via the de-repression of Rex-regulated proteins. Similarly, the inhibition of Rex has been observed in response to high levels of NO, a signaling molecule utilized during wound repair that is present at high levels in lung epithelial cells in patients with inflammatory diseases such as asthma or COPD [134,135,136]. Rex is also the major regulator of staphylococcal respiratory response AB genes (*srrAB*), a two-component system that regulates virulence factors and genes involved in anaerobic metabolism [137,138,139]. Mutations in *srrAB* allow for *S. aureus* SCVs to grow rapidly yet retain aminoglycoside resistance that was acquired during the SCV phase [140]. *S. aureus* glycolysis is also necessary for the upregulation of itaconate production, as demonstrated in a mouse pulmonary infection model. High itaconate levels in turn lead to the production of extracellular polysaccharide and biofilm formation [136].

Like bacterial cells, host cell metabolism also plays an important role in immune response. Hypoxia-inducible factor 1α (*hif1α*) is an important gene that controls both immune response and cellular metabolism [141]. HIF1α activation stimulates the production of pro-IL-1β, an important part of the inflammasome response, and promotes glycolysis. Importantly, HIF1α expression is induced during *S. aureus* infection of human cells, and the metabolic state of the bacterial cell plays an important role in this expression [141,142]. *S. aureus* mutants deficient in cellular glycolysis are unable to stimulate host cell HIF1α expression and therefore host cell glycolysis [141]. Contrarily, *S. aureus* SCV infection stimulates more host glycolysis than wild-type *S. aureus* infection, an effect that has been demonstrated in multiple host cell types [142]. Bacterial glycolysis appears to also play an important role in establishing infection, an effect that has been demonstrated using an in vivo mouse cutaneous infection model. Mice that were infected with glycolysis-deficient *S. aureus* demonstrated a significantly lower bacterial burden than those infected with wild-type strains [141]. These findings highlight the complex and important interplay between bacterial and host metabolic processes. 

Host cell anaerobic metabolism has an important secondary cellular consequence, the induction of necroptosis pathways [142]. Necroptosis is a type of host cell death that, unlike apoptosis, releases viable bacteria, thus promoting bacterial persistence [143]. Necroptosis is further upregulated by SCVs compared to wild-type *S. aureus* and is not activated when host cells are infected by glycolysis-deficient *S. aureus*. An in vivo mouse model demonstrated that mice unable to utilize necroptosis had greater *S. aureus* persistence compared to wild-type mice. This pathway provides an important mechanistic link between bacterial anaerobic metabolism and infection persistence. 

Another notable effect of anaerobic metabolism is the inhibition of trained immunity. Trained immunity is the process wherein the innate immune system develops memory of an infection and is therefore strengthened against re-infection with the same organism [144]. Unlike the adaptive immune response, this memory is thought to be accomplished through a series of epigenetic changes and is highly regulated by the amount of intracellular fumarate [145,146]. Fumarate is a TCA cycle substrate that induces both the trained immunity response and glycolysis [147]. Infection with *S. aureus* SCVs leads to lower amounts of intracellular fumarate than infection with wild-type strains, an effect that is mediated by an increase in *fumC* activity, an enzyme that breaks down fumarate [142]. Therefore, SCVs do not induce trained immunity to the degree of wild-type *S. aureus*, as validated using an in vivo mouse model. Even in the presence of a mixed SCV and wild-type population, the excess *fumC* activity from the SCV isolates prevents the trained immunity response. Of note, non-SCV strains of S. aureus also can enhance the production of FumC [148,149]. This increases susceptibility to infection upon future *S. aureus* exposure.

## 9. Intracellular *S. aureus* Is More Resistant to the Effects of Antibiotics through Multifaceted Mechanisms

**Figure 3:**
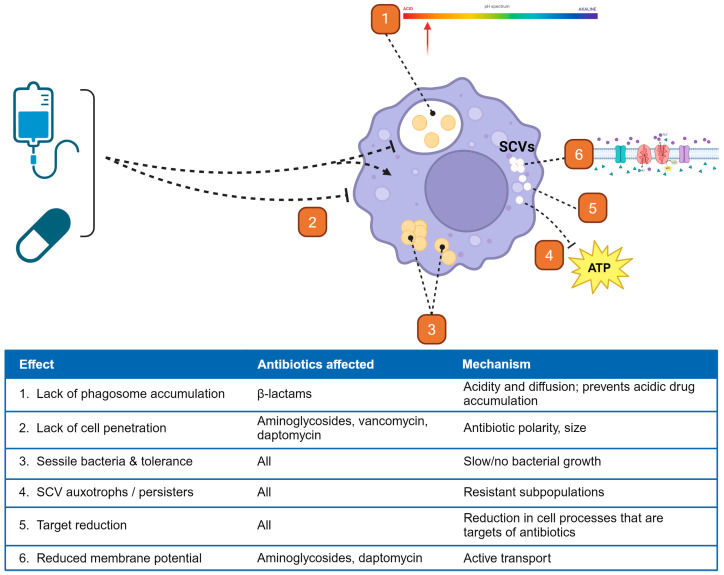
Mechanisms of reduced antibiotic activity against intracellular *S. aureus*.

The ability for *S. aureus* to survive in the intracellular environment provides protection from many antibiotics. These mechanisms are outlined in Figure 3. First, most antibiotics are either unable to penetrate the intracellular space or lack sufficient penetration for microbial killing. For example, β-lactams, an important class in the clinical management of *S. aureus* infections, do not accumulate within phagocytes, despite their ability to diffuse through membranes [150]. This is thought to be due to their weak acidity, which leads to lower accumulation within the already acidic intracellular space [151]. Aminoglycosides are unable to cross the membrane due to their polarity but have been noted to enter the intracellular space by endocytosis [152]. This process requires a longer duration of drug exposure and ultimately leads to localization within lysosomes. However, optimizing aminoglycoside exposures for this effect is limited by their well-known toxicities, namely nephrotoxicity and ototoxicity [153]. Conversely, lincosamides (clindamycin), macrolides, and fluoroquinolones all demonstrate accumulation within the intracellular space [150].

Interestingly, the intracellular accumulation of antibiotics does not always correlate with the antibiotic’s activity within that space. One study evaluating the intracellular activity of a variety of antibiotics found that intracellular activity was consistently lower than extracellular, despite the high intracellular accumulation of some of the antibiotics used [154]. Overall, the extent of antibiotic activity was greatly dependent on both the concentration and time of antibiotic exposure for all agents. This disconnect between intracellular concentration and activity points to other important factors that affect antibiotic activity within the intracellular environment.

Intracellular bacteria undergo several changes that lead to increased antibiotic resistance. In SCVs, the pattern of antimicrobial resistance is dependent on the specific auxotrophic type. Hemin or menadione auxotrophic SCVs are typically resistant to aminoglycosides [40]. The inhibition of electron transport within these cells leads to a reduction in the electrochemical gradient across the membrane. This gradient is required for the uptake of aminoglycosides into *S. aureus*. The other type of SCV, thymidine auxotrophs, are resistant to sulfa antibiotics (trimethoprim–sulfamethoxazole) as a result of disruption of the tetrahydrofolic acid pathway [155]. Thymidine auxotrophy results in reduced ClpC activity, which is needed to activate aconitase, an enzyme involved in the intraconversion of the tricarboxylic acid citrate, *cis*-aconitate, and isocitrate in the TCA cycle [156]. Hence, the reduced TCA activity results in an SCV similar to menadione and hemin auxotrophs [155,157]. The slow-growing SCV phenotype also leads to decreased susceptibility to cell wall-active antibiotics, such as β-lactams and vancomycin [158].

Because all three SCV auxotrophic classes lead to disruption of the electron transport chain, SCVs are deficient in ATP. This ATP depletion has been linked to the formation of persister cells from exponential-phase normal-colony phenotype *S. aureus* [159]. An in vitro study found that persister cells arise due to a stochastic change from the exponential to stationary growth phase, a change that was accompanied by a large decrease in ATP levels. This change to the stationary phase was associated with a 100- to 1000-fold increase in survival after antibiotic challenge. Another study found that the inactivation of enzymes involved in the TCA cycle (*sucA* or *sucB*) increased the formation of persister cells in stationary-phase cultures [65]. Interestingly, this study did not find a consistent decrease in ATP levels amongst persister cells formed during the stationary phase, but it did note lower membrane potential. This effect was confirmed pharmacologically using a proton motive force inhibitor, which increased the formation of persister cells. As noted earlier, tomatidine and related derivatives inhibit bacterial ATP synthase (F_0_F_1_ATPase), which specifically kills *S. aureus* SCVs [54]. This selectivity in *S. aureus*, and not mammalian cells, points to the use of F_0_F_1_ATPase in auxotrophic SCVs to create a membrane gradient despite low ATP [160]. The inhibition of this enzyme is fatal for *S. aureus* as it collapses the membrane potential but leaves untargeted host cells unharmed [55].

Peyrusson et al. demonstrated that intracellular *S. aureus* persisters are produced because of antibiotic exposure. These bacteria exist in a non-dividing state and display activation of the stringent response and other stress responses [64]. The decreased metabolic state associated with intracellular persistence leads to the downregulation of many cellular processes that are the targets of bactericidal antibiotics, such as fluoroquinolones, aminoglycosides, and β-lactams, and causes widespread antibiotic tolerance. Collectively, the intracellular host environment along with the altered *S. aureus* metabolism (through multiple mechanisms) required to survive in this environment dramatically reduces the effect of common antibiotics used in bacteremia treatment, setting the stage for treatment failure.

## 10. Conclusions

Mortality from SAB has remained unacceptably high over the course of decades of improvements in diagnostics and therapeutics. In this review, we have summarized evidence on the role of tolerant and persistent populations of *S. aureus* compounded by their proclivity for anaerobic metabolism and intracellular invasion to escape host recognition and antibiotic treatment. This evidence suggests that intracellular *S. aureus* is a significant contributor to persistent bacteremia and treatment failure in patients. Current therapeutics either lack activity against tolerant and persistent populations due to their inability to kill quiescent cells and/or lack sufficient host cell penetration to eliminate intracellular *S. aureus*. Further mechanistic understanding of *S. aureus* intracellular invasion combined with targeting these bacterial populations for therapeutic treatment will be essential to reducing the prevalence of persistent SAB among patients.
